# Public attitudes toward COVID-19 misbehaviors: Perceived seriousness of the misbehavior and perceived severity of the appropriate punishment

**DOI:** 10.3389/fpsyg.2023.1177696

**Published:** 2023-06-14

**Authors:** Inna Levy, Keren Cohen-Louck, Sergio Herzog

**Affiliations:** ^1^Department of Criminology, Ariel University, Ariel, Israel; ^2^Zefat Academic College, Zefat, Israel

**Keywords:** attitudes, violation, COVID-19 misbehavior, COVID-19, reckless behaviors

## Abstract

**Purpose:**

This interdisciplinary study explores attitudes toward health-related misbehaviors from a criminological point of view by comparing attitudes toward COVID-19 misbehaviors to the attitudes toward reckless behaviors related to driving and Human Immunodeficiency Virus (HIV) patients’ sexual behavior and identifying the predictors of attitudes toward COVID-19 misbehaviors.

**Methods:**

An online factorial survey included 679 respondents aged 18–89  years. The participants read various scenarios related to the violation of COVID-19 restrictions, reckless sexual behavior among HIV patients, and reckless driving. The participants evaluated the seriousness of each behavior and the appropriate severity of the punishment in each scenario. Within the scenarios about COVID-19 misbehaviors, we manipulated such variables as the type of COVID-19 misbehavior and violators’ gender, ethnicity, and religiosity. Additionally, participants answered questions about their demographic characteristics, vaccination, fear of COVID-19, and perceived contribution of COVID-19 misbehaviors to COVID-19-related morbidity.

**Results:**

The results indicated that participants perceived COVID-19 misbehaviors as less serious (*Mean* = 8.11, *S.D.* = 2.49) and deserving a less severe punishment (*Mean* = 7.57, *S.D.* = 2.59) than reckless driving (*Mean* = 9.36, *S.D.* = 1.25; *Mean* = 9.09, *S.D.* = 1.30; respectively). Additionally, the key factor predicting public opinion regarding COVID-19-related misbehaviors was the perceived contribution of these misbehaviors to virus-related morbidity. The perceived contribution to morbidity explained 52% of the variance in the seriousness of misbehavior and 53% of the severity of appropriate punishment.

**Conclusions:**

The findings suggest that it is critical to advocate for and reinforce the public’s understanding of the association between the increase in morbidity and the violation of restrictions preventing the transmission of viruses. Our findings also support the notion that the definitions of “crime” and “deviance” are not inherent or intrinsic but are created by the social context.

## Introduction

During the first stages of the COVID-19 outbreak, in the absence of vaccines and cures, governments issued restrictions to contain the virus, including stay-at-home orders (lockdowns), restricted citizens’ travel movements, limited and prohibited mass gatherings, enforced business shutdowns, and mandated mask-wearing (Chernozhukov et al., 2021; Clair et al., 2021). Although these containment measures effectively reduce virus transmission (e.g., Chernozhukov et al., 2021), many citizens unintentionally or deliberately violated COVID-19-related restrictions (Harris, 2020). Thus, because deviant behavior is *inter alia* a byproduct of rule-creation (Becker, 1963), the establishment of COVID-19 restrictions created a new type of deviant behavior—COVID-19-related misbehavior. However, there are no studies on public attitudes toward COVID-19 misbehaviors.

It is critical to research factors predicting public attitudes toward COVID-19 misbehaviors from a criminological perspective on public punitiveness because attitudes toward law violations and misbehaviors, in general, tend to shape policies and practices (Roberts and Stalans, 2018). Understanding attitudes toward law violations and misbehaviors is an important criterion for allocating resources and identifying priorities in crime control and prevention (e.g., Sherman et al., 2016) and for deciding whether to criminalize particular conduct (Ashworth, 2009). The current study addresses the gap in knowledge regarding public attitudes toward COVID-19 misbehavior by comparing these attitudes to the public attitudes toward reckless behaviors related to driving and Human Immunodeficiency Virus (HIV) patients’ sexual behavior. Furthermore, this research examines the association between attitudes toward COVID-19 misbehavior, situational factors (e.g., type of misbehavior, characteristics of the violators), and the characteristics of the violators and respondents.

### COVID-19 misbehaviors in the context of reckless behaviors

Reckless behaviors represent situations where people risk others’ lives and health by disregarding existing rules and laws (Erev et al., 2020; Roth et al., 2020). COVID-19 misbehavior is a type of reckless behavior (e.g., Harris, 2020) because the violation of COVID-19 restrictions imposed a high cost on public health and well-being by increasing self- and others’ risk of contamination (Ahmed et al., 2021). To better understand public attitudes toward COVID-19 misbehaviors, it is essential to compare attitudes toward this new type of reckless behavior to attitudes toward other reckless behaviors, such as reckless driving and reckless sexual behavior among HIV patients.

Similar to COVID-19 misbehaviors, reckless driving and reckless sexual behavior among HIV patients represent situations in which people endanger others’ lives and health by violating laws and norms (Erev et al., 2020; Roth et al., 2020). Drivers who disregard traffic laws (e.g., texting during driving, making an illegal turn) deviate from safe driving (Malta, 2004), and place themselves and other drivers and/or their passengers at risk of physical injury or mortality (Patil et al., 2006). Likewise, HIV-positive individuals who engage in unprotected intercourse violate laws requiring people living with HIV to disclose their HIV status before engaging in sexual intercourse or exchanging needles (Cameron, 2009; Novak, 2021) and therefore risk the transmission of HIV (Munro, 2007).

As in reckless driving and reckless sexual conduct among HIV patients, in cases of COVID-19 misbehaviors, health-related damages are not a definite result of rule violation, but the danger of such damages can be foreseen. However, compared to traffic laws and laws regarding the reckless transmission of HIV, the COVID-19-related restrictions represented a relatively new set of rules. Due to the novelty of COVID-19, COVID-19-related restrictions have been surrounded by controversy, and some people do not believe that COVID-19 is a dangerous disease (Siegrist et al., 2021); some even think that it is a hoax that poses no threat at all (Cohen-Louck and Levy, 2021).

The very fact that these COVID-19-related “regulations” or “restrictions” are referred to as such and as not “criminal laws” suggests that they do not have the same legally binding power. Conversely, the dangers of damaged health and mortality due to reckless driving and reckless transmission of HIV are well established, and there are criminal laws prohibiting these types of reckless behaviors (Cameron, 2009; Novak, 2021). Thus, considering that consensus regarding norms and values is associated with more negative attitudes toward offenses (e.g., Wenzel et al., 2021), it is possible to assume that violations of relatively established rules of behavior (e.g., traffic laws) will be associated with more negative public attitudes than violations of novel rules (COVID-19 restrictions). The hypothesis is as follows:

*H1*: Attitudes toward misbehaviors regarding more established norms of behavior (reckless driving and HIV patients’ reckless sexual behavior) will be more negative than attitudes toward misbehaviors regarding relatively new norms (COVID-19 misbehavior).

### Situational characteristics of COVID-19 misbehavior

The key situational factors influencing public attitudes toward crimes are the type of crime and severity of harm. The research on crime type indicates that people tend to express more negative attitudes toward violent crimes than nonviolent crimes (e.g., Perkins et al., 2009; Hardcastle et al., 2011; Herzog, 2017; Adriaenssen et al., 2020). Also, public support for harsher punishments is higher regarding violent offenses such as murder and rape than white-collar and victimless crimes (e.g., Einat and Herzog, 2011; Adriaenssen et al., 2020; Levy and Cohen-Louck, 2021). Furthermore, public support for capital punishment is higher in murder, terrorism, and sexual abuse cases than in other offenses (e.g., Qi and Oberwittler, 2009; Cohen-Louck et al., 2021; Dierenfeldt et al., 2021). As for perceived crime severity and harmfulness, attitudes tend to be more negative in crimes rated as more severe (Levy et al., 2020; Cohen-Louck et al., 2021) and involving physical harm than psychological or financial harm (Levy et al., 2020, e.g., Herzog, 2008; Levy and Kerschke-Risch, 2020; Levy and Rozmann, 2021).

Although there is no research on attitudes toward different types of COVID-19 misbehavior, it is reasonable to assume that some COVID-19 violations endanger public health more than others. For example, a quarantine violation by a confirmed COVID-19 patient or an individual who had contact with a confirmed COVID-19 patient has a higher risk for public health than a healthy individual who does not wear masks in public or attend mass gatherings. Based on the findings regarding crime type and severity, we hypothesize the following:

*H2*: There is a significant association between attitudes toward COVID-19 misbehaviors and the type of misbehavior. COVID-19 misbehaviors that represent a greater danger to public health (misbehaviors involving confirmed COVID-19 patients) will be associated with more negative public attitudes than misbehaviors with a lesser danger to public health (COVID-19 misbehaviors by healthy individuals).

*H3*: There is a significant positive association between perceptions of COVID-19 misbehavior contribution to COVID-19 morbidity and attitudes toward these misbehaviors. High levels of perceived contribution to COVID-19 morbidity will be associated with more negative attitudes.

Additional situational factors associated with attitudes toward crimes are perpetrators’ demographic characteristics (e.g., Fishman et al., 2006; Rozmann and Levy, 2019; Lehmann et al., 2020; Levy et al., 2020; Ulmer et al., 2020; Cohen-Louck et al., 2021; Levy and Rozmann, 2021), and this study focused on the gender, ethnicity, and religiosity of COVID-19 restriction violators. Violator gender may affect attitudes toward COVID-19 because the public tends to judge crimes committed by women as less serious than crimes committed by men (Herzog, 2008, e.g., de Vogel and de Spa, 2019). Regarding ethnicity, attitudes toward offenders affiliated with minority groups, such as African Americans and Latinos, are more negative and punitive than attitudes toward White offenders (Lehmann et al., 2020; Ulmer et al., 2020). Similarly, the Israeli public tends to judge Arab offenders more harshly than Jewish offenders (Levy and Rozmann, 2021, e.g., Fishman et al., 2006; Rozmann and Nahari, 2021). Regarding religiosity, it is possible that violator religiosity will affect public attitudes toward COVID-19 misbehaviors since ultraorthodox Jewish communities were blamed for the spread of COVID-19 (Gilman, 2021). The hypotheses were as follows:

*H4*: There is a significant association between attitudes toward COVID-19 misbehaviors and violator gender, ethnicity, and religiosity: Participants will express more negative attitudes toward COVID-19 misbehaviors committed by male, Arab, and ultraorthodox violators than by female, Jewish, and secular/traditional violators.

### Participants’ characteristics

Attitudes toward COVID-19 misbehaviors may also be associated with participants’ characteristics because attitudes toward crimes and perpetrators are associated *inter alia* with participants’ demographic characteristics (Rozmann and Levy, 2019; Levy et al., 2020; Cohen-Louck et al., 2021; Levy and Rozmann, 2021). Thus, older people generally rate crimes as more serious (Adriaenssen et al., 2019, 2020) and tend to hold more punitive attitudes than younger people (Payne et al., 2004; Frost, 2010). Individuals with higher levels of religiosity and ethnic minorities rate crimes generally as more serious (Rossi et al., 1974; Adriaenssen et al., 2019) and are more punitive (Levy and Reuven, 2017).

Regarding gender, some studies found that women rated crimes as more serious than did men (e.g., Schoepfer et al., 2007; Adriaenssen et al., 2019), while others did not find an association between gender and attitudes toward crimes (Leeper Piquero et al., 2008). However, regarding punitiveness, many studies indicate that men support harsher punishments than women (e.g., Anderson et al., 2017; Adinkrah and Clemens, 2018; Godcharles et al., 2019). Since women perceive COVID-19 as a more serious health problem than men (Galasso et al., 2020), it is possible to assume that women will express more negative attitudes toward COVID-19 misbehaviors.

Additional participants’ characteristics that may affect attitudes toward COVID-19 misbehaviors are individual fears of COVID-19. The research on fear of COVID-19 focused on psychological effects (e.g., Qiu et al., 2020; Braun-Lewensohn et al., 2021) and did not address the effects of fear of COVID-19 on attitudes toward COVID-19 misbehaviors. However, since people with high levels of fear of crime tend to perceive crimes as more serious and tend to be more punitive (e.g., Dowler, 2003; Klama and Egan, 2011), it is possible to assume that fear of COVID-19 and attitudes toward COVID-19 are associated. Based on these findings, we hypothesize the following:

*H5*: There is an association between attitudes toward COVID-19 misbehaviors and participants’ demographic characteristics: Participants who are female, older, religious, and belong to the ethnic minority group will express more negative attitudes toward COVID-19 misbehaviors than will participants who are male, younger, secular and belong to the ethnic majority group.

*H6*: There is a positive association between fear of COVID-19 and attitudes toward COVID-19 misbehaviors: High levels of fear of COVID-19 will be associated with more negative attitudes toward COVID-19 misbehaviors.

### Current research

This study aimed to compare attitudes toward COVID-19 misbehaviors to attitudes toward reckless driving and reckless HIV-related sexual behavior. Moreover, this study identifies the factors predicting two aspects of public attitudes toward COVID-19 misbehaviors: perception of the seriousness of the misbehavior and the appropriate severity of punishment. This study is unique because it refers to a health-related issue of COVID-19 misbehaviors from a criminological perspective. Also, this study adopted an integrative ecological framework (McLaren, 2005) that assumes that attitudes are affected by the interaction between individual (respondent-related variables) and contextual (situational) factors (McLaren, 2005; Espelage et al., 2013; Levy and Reuven, 2018).

This study employed a factorial survey design to explore the combined effects of situational characteristics and participants’ characteristics on attitudes toward COVID-19 misbehaviors. The factorial survey combines a controlled, randomized quasi-experimental design with a representative sampling of a conventional survey (Herzog, 2003; Herzog, 2017). The complexity of the research design and the manipulation of multiple factors reduce social desirability effects on the participant’s judgments (Wallander, 2009). The findings of this study may be useful to law and policymakers for determining punishment thresholds for the reckless behavior of COVID-19 instructions.

## Methods

### Participants

The online survey included 679 respondents. The age range was 18–89 years (*Mean* = 38.46, *S.D.* = 13.90), and approximately half of the respondents were male (49.8%). All participants spoke Hebrew fluently. The majority of the respondents were Jewish (87.3%), 10.9% Arab, and approximately 1.8% Druze. More than half (53%) of the respondents were married, 39% single, 6.6% divorced, and 1.3% widowed. Approximately 50.5% of respondents defined their household economic status as lower than average, 30.3% defined it as average, and approximately 19.2% defined it as higher than average. In terms of religiosity, 47.7% of the respondents defined themselves as secular, 32.7% as traditional, and 19.6% as religious. Regarding political affiliation, the majority (48.7%) reported holding leftist political views, 17.7% rightist political views, and 33.6% centrist political views.

### Measurements

#### Scenarios

We used scenarios to manipulate the type of misbehavior and violators’ characteristics. The scenarios described individuals who violated COVID-19 regulations regarding masks, quarantine and participation in multiparticipant events (wedding/funeral/party/protest), individuals infected with HIV having unsafe sex, and individuals engaged in reckless driving (reading texts while driving/illegal turning). The violators’ characteristics included gender (man/woman), ethnic affiliation (Arab/Jew), and religiosity (secular/religious/orthodox).

#### Misbehavior seriousness

Respondents evaluated the seriousness of different misbehaviors presented in the scenarios on a scale from “1” (not at all severe) to “10” (very severe).

#### The severity of appropriate punishment

Respondents evaluated the appropriate severity of the punishment in each scenario in response to the following question: “What should be the appropriate severity of punishment in this case?” The answers ranged from “1” (not at all severe) to “10” (very severe).

#### Contribution to the increase in morbidity

The participants assessed the extent to which individuals in COVID-19 scenarios contributed to the morbidity increase on the scale from “1” (not at all) to “10” (a very large extent).

#### Fear of COVID-19

We used the fear of COVID-19 scale (Ahorsu et al., 2020), which assesses reactions to the pandemic through items such as “I am most afraid of COVID-19” and “I am afraid of losing my life because of COVID-19.” The scale includes seven items on a Likert-type scale ranging from “1” (strongly disagree) to “5” (strongly agree). The scale was translated into Hebrew and validated by Tzur Bitan et al. (2020). Cronbach’s alpha in the current study was 0.90.

#### Respondent demographic characteristics

In the last section of the survey, the respondents stated their gender, age, education, ethnicity (Arab, Druze, or Jew), family status (single/married/divorced/widowed), household economic status (low/average/high), religiosity (secular/traditional/religious), and political affiliation (leftist/rightist/centrist). We also included variables related to COVID-19 experiences and asked respondents whether they have experienced individual quarantine (yes/no); whether they have been infected with COVID-19 (yes/no); and whether they have been vaccinated against COVID-19 (no, for medical reasons/no, for nonmedical reasons/only one dose and waiting for the second dose/only one dose and do not intend to get the second dose/vaccinated with two doses).

### Procedure

#### Scenario sampling

This study adopted the factorial approach (see Herzog, 2017). Based on this approach, the chosen scenarios represent a random sample of scenarios from the population of all possible scenarios, based on the combination of all values of all research variables (see Wallander, 2009; Su and Steiner, 2020). Thus, the random selection of values from the many factorial variables and the control of respondent personal characteristics (Rossi and Berk, 1997) facilitates unbiased estimations of the independent variables’ influence on respondent judgments (Dülmer, 2016; Herzog, 2017).

#### Data collection and ethics

The Ariel University Ethics Committee approved this study. The online survey for this study was conducted between 15.6.2021 and 26.6.2021. At the beginning of the survey, the questionnaires stated that participation in this study was anonymous, the responses were confidential, and the data would serve only research purposes. All respondents gave their informed consent to participate in this study. Each respondent addressed four scenarios. To minimize potential biases, we kept the questionnaire’s language as simple as possible (short and without professional jargon and terms). Before conducting the survey, we conducted a pretest to ensure the questionnaire’s clarity, obtained an initial test of measure reliability, and tested any unexpected response patterns (none were found).

The survey was conducted using the GeoCartography Knowledge Group (geokg) online panel called Panel4All, recognized as one of the largest internet panels in Israel, consisting of 130,000 potential respondents. In order to participate in surveys conducted on Panel4All, potential respondents must register on the panel’s website. During registration, individuals must provide information about their personal and household demographics and socio-economic and lifestyle details. The panel represents a diverse range of individuals, including native Israelis and new immigrants, secular and religious individuals, Jews and Arabs, men and women, and people of different age groups. To ensure the accuracy and validity of the data obtained from the surveys, the survey company ensures that the panel consists only of active participants, verifies the identity of panel members, and eliminates any duplicate registrations within the panel.

To guarantee the survey’s correct visual presentation, the participants could answer the survey only via personal computers. The sampling from the panel was random and based on a matrix created by a combination of the following variables: living area (area code), gender, age, ethnicity, and religiosity level. The various combinations of these variables’ values create small groups of compound characteristics. For example, there is a group of respondents who are “female, age 21–25, secular, from 08 area code (South Israel)” and a group of respondents who are “female, age 21–25, secular, from 04 area code (North Israel).” For each group, there is a specific quota of respondents. When the quota is reached, there is no further sampling of respondents with such characteristics.

This method enables the creation of samples that match the characteristics of Israeli populations. To sample 679 participants, the survey company sent 6,000 invitations to the individuals listed on this panel. The panelists are invited to participate in the survey through email notifications informing them that a survey has been uploaded to the panel website. The invitation email includes a link that directs the panelists to the designated panel website. In order to access the survey, panelists must log in using their assigned username and password, ensuring secure and authenticated access to the survey platform. In online studies, those respondents who answer quickly are included in the study. Thus, out of 6,000 invitations, only the first 679 respondents had a chance to participate. Those who did not participate cannot be compared to potential participants who refused to answer a phone/door-to-door survey. They may have been slower and might have responded later if the slots were not filled. Therefore, the issue of nonresponse bias is irrelevant to this type of sampling.

Moreover, there are only modest differences in outcomes between samples with high and low response rates (Curtin et al., 2000; Fosnacht et al., 2017). The decision to sample approximately 679 respondents addressed that the Israeli population is close to 9 million, with a 95% confidence level and a 4.4% confidence interval. An understanding of Hebrew was among the inclusion criteria. Generally, the sample’s sociodemographic characteristics are similar to the official distribution of these variables in the Israeli population when the survey was conducted (Central Bureau of Statistics, 2021).

#### Data analysis

All analyses were carried out using SPSS Version 25. To assess the differences in attitudes toward various misbehaviors, we conducted MANCOVA and included the seriousness of misbehavior and severity of appropriate punishment as dependent variables. MANCOVA and Pearson’s correlation was used to assess the association between attitudes toward COVID-19 misbehavior and the association between predicting variables. Within the MANCOVA, we controlled for demographic and pandemic-related variables that were associated with attitudes toward COVID-19 misbehaviors (Table 1): participants’ gender (0 = female, 1 = male), age, ethnicity (Arab = 0, Jewish = 1), secular (secular = 1, all else = 0), religious (religious = 1, all else = 0), exposure to COVID-19 quarantine (yes = 1, no = 0), and vaccination (yes = 1, no = 0). Additionally, we used hierarchical multiple regressions to assess the integrative model. We included demographic variables significantly associated with attitudes toward COVID-19 misbehavior within the regressions.

**Table 1:** Differences in attitudes toward COVID-19 misbehavior by participants’ demographic characteristics.

	Attitudes toward COVID-19 Misbehavior					
	Seriousness of misbehavior		Severity of appropriate punishment		MANOVA	
	Mean	S.E.	Mean	S.E.	*F*	df
**Participants’ demographic characteristics**						
Gender						
Women	6.99	0.12	6.31	0.12	4.83^***^	2, 1,269
Men	6.48	0.12	5.82	0.12		
*F* (1, 1,272)	9.46^**^		8.41^**^			
*η^2^*	0.01		0.01			
**Family status**						
Single	6.50	0.13	5.76	0.14	2.44^*^	6, 2,518
Married	6.86	0.13	6.21	0.12		
Divorced	7.33	0.33	6.91	0.33		
Widowed	7.20	0.93	6.10	0.95		
*F* (1, 1,263)	2.61^*^		4.36^**^			
*η^2^*	0.01		0.01			
**Ethnicity**						
Arabs	7.12	0.24	6.68	0.24	4.70^**^	2, 1,269
Jews	6.68	0.09	5.97	0.09		
*F* (3, 1,268)	3.11		7.67^**^			
*η^2^*	0.00		0.01			
**Religiosity**						
Secular	6.62	0.13	5.83	0.13	3.26^**^	6, 2,190
Traditional	6.91	0.16	6.34	0.16		
Religious	7.11	0.26	6.36	0.27		
Orthodox	5.89	0.31	5.09	0.32		
*F* (1, 1,112)	3.89^**^		5.50^**^			
*η^2^*	0.00		0.02			
**Political affiliation**						
Right	6.76	0.12	6.05	0.12	1.74	4, 2,534
Center	6.85	0.14	6.28	0.15		
Left	6.42	0.20	5.68	0.20		
*F* (3, 1,268)	1.59		2.84			
*η^2^*	0.00		0.00			
**Have experienced COVID-19 quarantine**						
No	6.70	0.11	6.13	0.11	3.88^*^	0.01
Yes	6.78	0.14	5.95	0.14		
*F* (1, 1,272)	0.18		0.96			
*η^2^*	0.00		0.00			
**Have been diagnosed with COVID-19**						
No	6.77	0.09	6.12	0.09	2.60	2, 1,269
Yes	6.39	0.27	5.53	0.27		
*F* (1, 1,270)	1.82		4.32^*^			
*η^2^*	0.00		0.00			
**Have been vaccinated against COVID-19**						
No	5.98	0.21	5.51	0.21	8.47^***^	2, 1,269
Yes	6.87	0.09	6.17	0.09		
*F* (1, 1,270)	15.50^***^		8.04^**^			
*η^2^*	0.01		0.01			
**Educational level**						
High school	6.75	0.13	6.12	0.14	0.98	6, 2,534
Professional certificate	6.37	0.21	5.75	0.21		
B.A.	6.88	0.15	6.20	0.15		
M.A.+	6.76	0.22	5.96	0.23		
*F* (3, 1,268)	1.34		1.12			
*η^2^*	0.00		0.00			

^*^p < 0.05, ^**^p < 0.01, ^***^p < 0.001; S.E.= Standard error.

## Results

### Attitudes toward COVID-19 misbehavior and demographic characteristics

There was a significant weak and positive correlation between age and attitudes toward COVID-19 misbehavior: *r* = 0.07, *p* < 0.001 for the seriousness of COVID-19 misbehaviors and *r* = 0.10, *p* < 0.001 for the severity of appropriate punishment. The results of MANOVA (Table 1) showed a significant difference in attitudes toward COVID-19 misbehavior by gender, family status, ethnicity, religiosity, and vaccination against COVID-19. Women rank the seriousness of COVID-19 misbehaviors and the severity of appropriate punishment higher than men. Married and divorced participants ranked the seriousness of misbehaviors and the severity of appropriate punishment higher than single widowers. There was no significant difference between single and widowed participants, and there was no significant difference between married and divorced participants. Therefore, we recoded family status into two dichotomous variables: single (single = 1, all else = 0) and married (married = 1, all else = 0).

Regarding ethnicity, Arab participants ranked COVID-19 misbehaviors as more serious and thought punishments should be more severe than Jewish participants. As for religiosity, secular, traditional, and religious participants ranked the seriousness of COVID-19 misbehavior and the severity of appropriate punishment higher than orthodox participants. There were no significant differences between secular, traditional, and religious participants’ rankings. Therefore, we recoded this variable to include it in the regression into three dichotomous variables: secular (secular = 1, all else = 0); religious (religious = 1, all else = 0); and traditional (traditional = 1, all else = 0).

Finally, the participants who had been vaccinated against COVID-19 expressed more negative attitudes toward COVID-19 misbehavior than those who had not been vaccinated. There was no significant association between COVID-19 misbehavior and political affiliation, COVID-19 diagnosis, or educational level. We also considered the association between attitudes toward COVID-19 misbehavior and experiences of quarantine statistically nonsignificant based on the results of ANOVAs. Thus, we included in the regressions the following participants’ characteristics: gender, age, ethnicity, religiosity, and vaccination status. We did not include in the regression participants’ family status due to a significant and relatively strong association between age and family status: *r* = −0.59, *p* < 0.001 for single and *r* = 0.44, *p* < 0.001 for married.

### Attitudes toward reckless misbehaviors related to COVID-19, HIV, and driving

To examine the effect of the type of reckless act, we compared four groups of reckless acts (Table 2): reckless driving, reckless sexual behavior among HIV patients, misbehaviors involving confirmed cases of COVID-19, and participation in mass gatherings banned due to COVID-19 restrictions. MANCOVA indicated significant differences in public attitudes by the type of act [*F*(6, 2,826) = 68.28, *η^2^* = 0.13, *p* = 0.00].

**Table 2:** Perceptions of the seriousness of misbehavior and severity of punishment by type of misbehavior (*N* = 1,424).

Type of misbehavior	Seriousness of misbehavior		Severity of punishment	
	Mean	(S.D.)	Mean	(S.D.)
1. Reckless driving	9.36^3***4***^	(1.25)	9.09^3***4***^	(1.30)
2. HIV-related reckless behavior	8.75^4***^	(2.05)	7.98^4***^	(2.31)
3. COVID-19 confirmed cases	8.11^1***4***^	(2.49)	7.57^1***4***^	(2.59)
4. Participation in mass gathering	6.24^1***2***3***^	(2.98)	5.58^1***2***3***^	(2.99)
*F_ANOVA_*	118.91^***^		143.163^***^	
*df*	3, 1,414		3, 1,414	
*η* *^2^*	0.20		0.23	

^*^p < 0.05, ^**^p < 0.01, and ^***^p < 0.001. ^1^Reckless driving; ^2^HIV-related reckless behavior; ^3^COVID-19 confirmed cases (misbehavior involving a diagnosis of COVID-19 or contact with confirmed cases of COVID-19 = 1, all else = 0); ^4^Participation in mass gathering (wedding, party, funeral, and protest).

These results indicated that the participants perceived the reckless driving scenarios as significantly more serious and deserving harsher punishments than COVID-19-related misbehaviors; however, there were no significant differences in attitudes between the scenarios of reckless driving and the scenario of unsafe sexual behavior among individuals diagnosed with HIV. Additionally, there was no significant difference in attitudes between HIV-related reckless sexual behavior and COVID-19-confirmed case-related misbehaviors. Regarding the differences between the two groups of COVID-19 misbehaviors, the violations of restrictions on participation in mass gatherings were perceived as less negative.

As for the significant differences between specific COVID-19 misbehaviors [*F_MANCOVA_*(14, 2,120) = 11.91, *η^2^* = 0.07, *p* = 0.00], ANCOVA (Table 3) indicated that the participants ranked the scenarios involving COVID-19 confirmed patients or individuals who had contact with confirmed COVID-19 patients (scenarios 1–3, Table 3) as more severe and deserving a harsher punishment than the scenarios of unsafe COVID-19-related behaviors among individuals who were not described as infected or as having contact with infected individuals (scenarios 4–8, Table 3).

**Table 3:** Perceptions of the seriousness of misbehavior and severity of punishment by type of misbehavior (*n* = 1,075).

COVID-19 misbehavior	Seriousness of misbehavior		Severity of punishment	
	Mean	(S.D.)	Mean	(S.D.)
1. COVID-19 confirmed patient walked in a mall without a mask (*n* = 95)	8.34	(2.00)	7.83	(2.20)
2. A person who was in contact with confirmed COVID-19 patient walked in a mall without a mask (*n* = 95)	8.05	(2.44)	7.53	(2.56)
3. COVID-19 confirmed patient walked in a mall with a mask (*n* = 96)	7.94	(2.94)	7.34	(2.96)
4. An individual participated in a wedding with many attendees (*n* = 193)	6.89	(2.58)	6.07	(2.67)
5. An individual participated in a party with many attendees (*n* = 83)	6.65	(2.85)	5.91	(2.91)
6. An individual walked in a mall without a mask (*n* = 84)	6.47	(2.86)	5.81	(2.76)
7. An individual participated in a funeral with many attendees (*n* = 209)	6.39	(3.02)	5.60	(3.02)
8. An individual participated in a protest with many attendees (*n* = 194)	5.25	(3.12)	4.76	(3.13)
*F_ANCOVA_*	21.23^***^		22.25^***^	
*df*	7, 1,061		7,1,061	
*η^2^*	0.12		0.12	

^*^p < 0.05, ^**^p < 0.01, and ^***^p < 0.001.

### Prediction of attitudes toward COVID-19 misbehavior

The hierarchical multiple regressions for predicting attitudes toward COVID-19 misbehavior included four steps. Participants’ sociodemographic characteristics were entered in the first step, situational characteristics were entered second, fear of COVID-19 was entered third, and perceived contribution to the increase in morbidity was entered in the fourth step. Correlations among the predictor variables (Supplementary Table S1; Supplementary material) were weak to moderate, ranging from *r* = 0.06, *p* < 0.05 to *r* = 0.53, *p* < 0.00, thus indicating no multicollinearity. All models were significant (Table 4), with the explained variance being 67% for the seriousness of misbehavior and 69% for the severity of the appropriate punishment. The least contributing variables to predicting attitudes toward COVID-19 misbehavior were sociodemographic characteristics (4% of variance) and aspects of fear of COVID-19 (1–2% of variance). The situational characteristics explained 10% of the variance, and the strongest predictor was the perceived contribution to morbidity (regarding COVID-19 misbehaviors). The perceived contribution to morbidity explained 52% of the variance in the seriousness of misbehavior and 53% of the severity of appropriate punishment.

**Table 4:** Multiple regressions predicting attitudes toward COVID-19 misbehavior (*N* = 1,272).

	Seriousness of misbehavior			Severity of appropriate punishment		
	*B*	*S.E. B*	β	*B*	*S.E. B*	β
Step 1						
**Participants’ characteristics**						
Gender^1^	−0.58	0.16	−0.10^*^	−0.55	0.17	−0.09^**^
Age	0.01	0.01	0.06^*^	0.02	0.01	0.09^**^
Ethnicity^2^	−1.21	0.38	−0.14^**^	−1.55	0.39	−0.17^***^
Secular^3^	0.48	0.34	0.08	0.55	0.34	0.09
Traditional^4^	0.79	0.35	0.12^*^	1.04	0.35	0.16^**^
Religious^5^	1.05	0.41	0.11^*^	1.06	0.41	0.11^*^
Vaccination^6^	0.98	0.23	0.12^***^	0.76	0.24	0.09^**^
***R^2^_Adj_***	0.04^**^			0.04^***^		
Step 2						
**Participants’ characteristics**						
Gender	−0.56	0.16	−0.10^***^	−0.54	0.16	−0.09^**^
Age	0.01	0.01	0.06^*^	0.02	0.01	0.09^**^
Ethnicity	−1.16	0.36	−0.13^**^	−1.49	0.37	−0.16^***^
Secular	0.49	0.32	0.08	0.58	0.33	0.10
Traditional	0.80	0.33	0.12^*^	1.06	0.34	0.16^**^
Religious	0.95	0.39	0.10^*^	0.98	0.39	0.10^*^
Vaccination	1.04	0.23	0.13^***^	0.81	0.23	0.10^***^
**Situational characteristics**						
violator gender^7^	0.47	0.16	0.08^**^	0.50	0.16	0.08^**^
violator ethnicity^8^	−0.20	0.19	−0.03	−0.32	0.19	−0.05
secular violator^9^	0.22	0.18	0.04	0.21	0.18	0.03
orthodox violator^10^	0.004	0.22	0.001	−0.04	0.22	−0.01
Funeral^11^	−0.35	0.24	−0.04	−0.36	0.24	−0.05
Wedding^12^	0.23	0.24	0.03	0.28	0.25	0.03
Protest^13^	−1.43	0.25	−0.18^***^	−1.21	0.25	−0.14^***^
Confirmed COVID-19^14^	1.46	0.22	0.21^***^	1.62	0.22	0.23^***^
***ΔR^2^_Adj_***	0.10^***^			0.10^***^		
Step 3						
**Participants’ characteristics**						
Gender	−0.49	0.16	−0.08^**^	−0.44	0.16	−0.08^**^
Age	0.02	0.01	0.07^*^	0.02	0.01	0.10^***^
Ethnicity	−0.90	0.37	−0.10^*^	−1.17	0.37	−0.13^**^
Secular	0.37	0.32	0.06	0.43	0.33	0.07
Traditional	0.62	0.33	0.10	0.84	0.34	0.13^*^
Religious	0.87	0.38	0.09^*^	0.88	0.39	0.09
Vaccination	1.04	0.22	0.13^***^	0.82	0.23	0.10^***^
**Situational characteristics**						
Violator gender	0.49	0.15	0.08^**^	0.48	0.16	0.08^**^
Violator ethnicity	−0.21	0.19	−0.03	−0.32	0.19	−0.05
Secular violator	0.22	0.18	0.04	0.21	0.18	0.03
Orthodox violator	0.02	0.22	0.01	−0.03	0.22	−0.004
Funeral	−0.36	0.24	−0.05	−0.38	0.24	−0.05
Wedding	0.21	0.24	0.03	0.25	0.25	0.03
Protest	−1.50	0.25	−0.18^***^	−1.29	0.25	−0.15^***^
Confirmed COVID-19	1.40	0.22	0.20	1.56	0.22	0.22^***^
**Fear of COVID-19**	0.40	0.10	0.11^***^	0.48	0.10	0.13^***^
***ΔR^2^_Adj_***	0.01^***^			0.02^***^		
Step 4						
**Participants’ characteristics**						
Gender	−0.27	0.10	−0.05^**^	−0.21	0.10	−0.03^*^
Age	0.01	0.004	0.05^**^	0.02	0.003	0.08^**^
Ethnicity	0.07	0.23	0.01	−0.18	0.23	−0.02
Secular	0.02	0.20	0.003	0.09	0.20	0.01
Traditional	−0.01	0.21	−0.002	0.16	0.20	0.02
Religious	0.41	0.24	0.04	0.43	0.24	0.04
Vaccination	0.21	0.14	0.03	−0.05	0.14	−0.01
**Situational characteristics**						
Violator gender	0.26	0.10	0.04	0.26	0.10	−0.04^**^
Violator ethnicity	−0.07	0.12	−0.01	−0.17	0.12	−0.02
Secular violator	0.06	0.11	0.01	0.05	0.11	0.01
Orthodox violator	0.12	0.14	0.02	0.06	0.13	0.01
Funeral	−0.28	0.15	−0.04	−0.27	0.15	−0.03
Wedding	−0.29	0.15	−0.04	−0.27	0.15	−0.03
Protest	−0.87	0.15	−0.11^***^	−0.65	0.15	−0.08^***^
Confirmed COVID-19	0.38	0.14	0.05^**^	0.48	0.14	0.07^***^
**Fear of COVID-19**	−0.07	0.06	−0.02	−0.01	0.06	−0.004
**Contribution to morbidity**	0.78	0.02	0.77^***^	0.81	0.02	0.78^***^
***ΔR^2^_Adj_***	0.52^***^			0.53^***^		
***Total R^2^_Adj_***	0.67			0.69		
*F*	148.26^***^			164.87^***^		
*df*	17, 1,257			17, 1,242		

^*^p < 0.05, ^**^p < 0.01, and ^***^p < 0.001.

1 Participants gender (0 = female, 1 = male).

2 Ethnicity (Arab = 0, Jewish = 1).

3 Secular (1 = secular; all else = 0).

4 Traditional (1 = traditional; all else = 0).

5 Religious (1 = religious; all else = 0).

6 Vaccination (0 = have not been vaccinated, 1 = vaccinated).

7 Violator Gender (0 = female, 1 = male).

8 Violator Ethnicity (Arab = 0, Jewish = 1).

9 Secular Violator (1 = secular; all else = 0).

10 Orthodox Violator (1 = orthodox; all else = 0).

11 Funeral (participated in multi-participant funeral = 1, all else = 0).

12 Wedding (participated in multi-participant wedding = 1, all else = 0).

13 Protest (participated in multi-participant protest = 1, all else = 0).

14 Confirmed COVID-19 (misbehavior involving a diagnosis of COVID-19 or contact with confirmed case of COVID-19 = 1, all else = 0).

In the final model for predicting the seriousness of misbehavior, the significant contributors included participants’ gender and age, participation in a protest, misbehavior related to confirmed cases of COVID-19 (individuals with confirmed COVID-19 or who were in contact with confirmed COVID-19), and perceived contribution to morbidity. In the final model for predicting the severity of appropriate punishment, the significant contributors were almost similar, with one exception: the contribution of the violator’s gender was significant.

Regarding the nature of the correlations (Table 4), the seriousness of misbehavior was ranked higher by female and older participants in scenarios other than attending a protest and related to confirmed cases of COVID-19, and when the perceived contribution to an increase in morbidity was high. The severity of appropriate punishment was ranked higher by female and older participants. Also, participants ranked the severity of appropriate punishment higher for cases of female violators, scenarios other than attending a protest, and scenarios related to confirmed cases of COVID-19. Finally, participants ranked the severity of appropriate punishment higher when they perceived misbehavior as contributing to an increase in morbidity. Most correlations between attitudes toward COVID-19 misbehaviors and independent variables were weak, except for the correlation between attitudes toward COVID-19 misbehaviors and perceived contribution to increased morbidity.

## Discussion

This study examined attitudes toward COVID-19 misbehaviors by addressing the perceived seriousness of misbehavior and the severity of appropriate punishment. The comparison between attitudes toward reckless driving, reckless HIV-related sexual behavior and COVID-19 misbehaviors indicates that the public perceives all types of COVID-19 misbehaviors as less negative than reckless driving. This finding partially supports our assumption (H_1_) and prior findings indicating that public attitudes tend to be more negative toward violations of established criminal laws that are characterized by a stronger consensus (e.g., Wenzel et al., 2021).

However, our findings suggest that a consensus may not be the key factor affecting attitudes toward recklessness in the context of health-related behaviors, and the risk of being infected may be a more significant factor. Hence, the attitudes toward reckless health-related behavior by infected individuals (HIV or COVID-19) were similar and perceived as significantly more negative than misbehavior by *participating in mass gatherings* that did not involve infected individuals. Thus, similar to attitudes toward criminal offenses (e.g., Herzog and Einat, 2016; Adriaenssen et al., 2019), not all COVID-19 misbehaviors are considered equally serious and deserving of equally harsh punishment. These findings underline the significance of the context of reckless behaviors and the risk of harm. Future studies should examine the factors that distinguish between the context of traffic laws and health-related laws.

Another set of intriguing findings identifies the predictors of attitudes toward COVID-19 misbehaviors within the integrative models, which include participants’ characteristics, situational characteristics, fear of COVID-19, and perceived contribution to morbidity. The findings indicate that respondent perception of the misbehaviors’ contribution to morbidity due to COVID-19 was the main predictor of public attitudes toward COVID-19 misbehaviors. As hypothesized (H_3_), misbehaviors that were perceived as contributing more to COVID-19-related morbidity elicited more negative attitudes: they were perceived as more serious and deserving of more severe punishment. The significant role of the perceived contribution to morbidity corresponds with the public’s tendency to express more negative and punitive attitudes toward crimes that cause physical harm (as opposed to financial or emotional harm) and toward crimes that are considered more harmful (e.g., Einat and Herzog, 2011; Rozmann and Levy, 2019; Adriaenssen et al., 2020; Levy et al., 2020; Levy and Kerschke-Risch, 2020; Cohen-Louck et al., 2021; Levy and Cohen-Louck, 2021). Thus, similar to public attitudes toward conventional crimes, attitudes toward COVID-19 misbehaviors are associated with respondents’ appraisal of the physical harmfulness of these misbehaviors.

The significance of the harmfulness of the misbehavior also manifests in the findings regarding the situational characteristics (H_2_) as evident from the inter-ranking of COVID-19 misbehaviors. Participants expressed more negative attitudes toward the misbehaviors in which the risk of COVID-19 transmission was more apparent: violations conducted by confirmed COVID-19 patients or individuals exposed to a confirmed COVID-19 patient. The results of the comparison between rankings of the various cases also emphasize the predominance of the harmfulness of misbehaviors and further clarify the effect of the situational context on public attitudes toward COVID-19 misbehaviors.

Moreover, the rankings of cases involving participation in different types of mass gatherings indicate that the nature of the gathering affects public opinion, and the violation of COVID-19 restrictions by participation in a protest was perceived as less serious and deserving of a lesser punishment than other types of mass gatherings (wedding, funeral, and party). It is possible that in situations perceived as socially important and necessary (funerals and protests), the violation of laws and restrictions is perceived less negatively. Considering that COVID-19 lockdowns and restrictions imposed relatively severe limitations on individual freedoms (Cohen-Louck and Levy, 2021), perhaps, the right to protest was perceived as more precious and significant. Regarding the violators’ characteristics (H_4_), the models showed that in the context of COVID-19 misbehaviors, violators’ characteristics such as ethnicity, religiosity and gender are not influential predictors of public attitudes.

Among the participants’ characteristics (H_5_) and related variables, only gender and age were significant predictors. Participants’ ethnicity, religiosity (H_5_) and fear of COVID-19 (H_6_) did not contribute to the prediction of public attitudes toward COVID-19 misbehaviors. However, in line with prior studies (Galasso et al., 2020) and as hypothesized (H_5_), female and older respondents expressed more negative attitudes toward COVID-19 misbehaviors than male and younger participants. Although the associations were relatively weak, the results suggest that gender and age play a more significant role in health-related attitudes than other demographic characteristics. This pattern corresponds with women’s higher vulnerability to the impact of disasters (Neumayer and Plümper, 2007) and higher sensitivity to and interest in health-related information (Ek, 2015), as well as with evidence that older individuals are more vulnerable to the physical effects of COVID-19 (e.g., Sohrabi et al., 2020).

## Limitations and future research

This research has yielded interesting results regarding public attitudes toward COVID-19-related misbehaviors, but some limitations should be noted. First, although the sample resembles the Israeli general public, the finding may not represent the attitudes among citizens who do not understand Hebrew: some immigrants and some Israeli Arabs. Second, due to the cross-sectional nature of this study’s design, further research is necessary to detect causal pathways between attitudes toward COVID-19 misbehaviors and participants’ characteristics and contextual characteristics of the violations. Third, since attitudes toward crimes and norm violations may change based on cultural context (e.g., Levy and Adam, 2018; Levy and Kerschke-Risch, 2020; Levy and Berenson, 2022; Levy and Kerschke-Risch, 2022), future studies should examine our model within various cultural contexts. Furthermore, future studies may examine attitudes toward different types of COVID-19 misbehaviors or health-related norms and restrictions and compare them to other reckless behaviors (e.g., violation of workplace safety procedures, unsafe/illegal storing of weapons or toxic substances, engaging in rough play or sports in inappropriate settings, and driving under the influence of alcohol). Finally, the current study was conducted during COVID-19 restrictions and lockdowns. Surveys provide a “snapshot” of public opinion at a specific period (Connelly, 2016); therefore, public opinion regarding COVID-19 misbehaviors may change following developments and innovations in prevention (vaccine), treatment (invention of medicine) and management of the disease (abolition of the restrictions).

## Conclusion

The COVID-19 pandemic created a need for new norms and restrictions, which in turn created a new type of deviant behavior—violation of COVID-19 restrictions. This study identified the factors associated with attitudes toward this new type of deviance. The findings indicate that when COVID-19 restrictions were in force, the public perceived COVID-19 misbehaviors as serious and deserving of a relatively severe punishment. Additionally, it appears that the key factor predicting public opinion regarding COVID-19-related misbehaviors is the perceived contribution of these misbehaviors to virus-related morbidity. This study promotes an interdisciplinary perspective on public health issues by exploring attitudes toward COVID-19 misbehavior *via* a criminological point of view by focusing on factors associated with public punitiveness toward health-related misbehaviors and comparing reckless behaviors in the context of driving and health. Future research should expand our knowledge on factors affecting public attitudes toward deviancy in different contexts.

### Implications for policy and practice

This study’s findings are significant from a health communication standpoint. Effective health communication plays a critical role in promoting healthy behaviors and preventing the spread of diseases (Street and Finset, 2022). This finding could potentially inform the development of more effective health communication strategies in the context of viral pandemics. Thus, to increase public support and cooperation with restrictions aimed at preventing virus transmission, it is critical to advocate and reinforce the public’s understanding of the association between the violation of restrictions and the increase in morbidity. From the social deviancy standpoint, our findings regarding public attitudes toward COVID-19 misbehaviors emphasize and support the notion that definitions of “crime” and “deviance” are not inherent or intrinsic but are created by the social context (Ugwudike, 2015).

Today, COVID-19 restrictions are less necessary due to the development of vaccines and improvements in the treatment of COVID-19. Additionally, it seems that people have gotten used to living with the COVID-19 pandemic. However, history shows that COVID-19 is not the first pandemic, and humankind has dealt with various types of viruses in the past (for a review, see Moghadami, 2017). Thus, the question is not “Will a new pandemic occur in the future?” but “When will a new pandemic occur?” (Cohen-Louck and Levy, 2021). Therefore, it is important to learn the lessons provided by the COVID-19 pandemic, including the factors that may explain public attitudes toward violations of rules and restrictions aimed at preventing the spread of deadly viruses.

## Data availability statement

The raw data supporting the conclusions of this article will be made available by the authors, without undue reservation.

## Ethics statement

The studies involving human participants were reviewed and approved by Ariel University Ethics Committee. The patients/participants provided their written informed consent to participate in this study.

## Author contributions

IL served as lead for statistical analyses and contributed equally to conceptualization, data curation, investigation, methodology, project administration, writing—the original draft, writing—the review, and editing. KC-L and SH contributed equally to conceptualization, data curation, investigation, methodology, project administration, writing—original draft, and writing—review and editing, and served in a supporting role for statistical analyses. All authors contributed to the article and approved the submitted version.

## Conflict of interest

The authors declare that the research was conducted in the absence of any commercial or financial relationships that could be construed as a potential conflict of interest.

## Publisher’s note

All claims expressed in this article are solely those of the authors and do not necessarily represent those of their affiliated organizations, or those of the publisher, the editors and the reviewers. Any product that may be evaluated in this article, or claim that may be made by its manufacturer, is not guaranteed or endorsed by the publisher.
