# Supplementary material for: Public attitudes toward COVID-19 misbehaviors: Perceived seriousness of the misbehavior and perceived severity of the appropriate punishment
Source: Front Psychol. 2023 Jun 14;14:1177696. doi: 10.3389/fpsyg.2023.1177696 (PMC10301832; doi:10.3389/fpsyg.2023.1177696)
Supplement: Supplementary file 1 [file Table_1.docx]

**Supplement Materials**

Table S1

*Correlations between the independent variables*

|  | 1 | 2 | 3 | 4 | 5 | 6 | 7 | 8 | 9 | 10 | 11 | 12 | 13 | 14 | 15 | 16 | |  |
| --- | --- | --- | --- | --- | --- | --- | --- | --- | --- | --- | --- | --- | --- | --- | --- | --- | --- | --- |
| 1. Gender^1^ | 1 |  |  |  |  |  |  |  |  |  |  |  |  |  |  |  |  |  |
| 2. Age | .046 | 1 |  |  |  |  |  |  |  |  |  |  |  |  |  |  |  |  |
| 3. Ethnicity^2^ | .048 | -.005 | 1 |  |  |  |  |  |  |  |  |  |  |  |  |  |  |  |
| 4. Secular^3^ | .024 | .004 | .319^***^ | 1 |  |  |  |  |  |  |  |  |  |  |  |  |  |  |
| 5. Traditional^4^ | .007 | .010 | .239^***^ | -.533^***^ | 1 |  |  |  |  |  |  |  |  |  |  |  |  |  |
| 6. Religious^5^ | .087*** | .052 | .127*** | -.282*** | -.212*** | 1 |  |  |  |  |  |  |  |  |  |  |  |  |
| 7.Vaccination^6^ | .022 | .066^*^ | .222^***^ | .168^***^ | .063^*^ | .030 | 1 |  |  |  |  |  |  |  |  |  |  |  |
| 8. Violator Gender^7^ | -.019 | .021 | .004 | -.005 | -.019 | .044 | -.007 | 1 |  |  |  |  |  |  |  |  |  |  |
| 9. Violator Ethnicity^8^ | -.002 | -.007 | .014 | -.010 | .011 | .018 | .011 | -.004 | 1 |  |  |  |  |  |  |  |  |  |
| 10. Secular Violator^9^ | .019 | -.010 | -.003 | .031 | -.012 | -.033 | -.010 | -.009 | .118^***^ | 1 |  |  |  |  |  |  |  |  |
| 11. Orthodox Violator^10^ | -.030 | -.022 | .002 | .019 | -.019 | -.003 | .020 | .023 | .305^***^ | .453^***^ | 1 |  |  |  |  |  |  |  |
| 12. Funeral^11^ | -.004 | .013 | -.019 | -.001 | -.016 | .036 | .000 | .014 | -.012 | .041 | .013 | 1 |  |  |  |  |  |  |
| 13. Wedding^12^ | -.009 | -.026 | -.013 | -.017 | .030 | -.017 | -.021 | .021 | -.047 | .026 | .037 | -.189^***^ | 1 |  |  |  |  |  |
| 14. Protest^13^ | .002 | .030 | -.007 | .012 | -.008 | -.017 | .056^*^ | -.052 | -.054 | .060^*^ | -.036 | -.188^***^ | -.179^***^ | 1 |  |  |  |  |
| 15. Confirmed COVID-19^14^ | -.004 | .033 | -.042 | -.034 | -.017 | .034 | .011 | .012 | -.004 | .035 | .016 | -.240^***^ | -.229^***^ | -.228^***^ | 1 |  |  |  |
| 16. Fear of COVID-19 | -.162*** | -.089^**^ | -.099^***^ | .016 | .063^*^ | -.099*** | .034 | .010 | -.012 | -.030 | -.003 | -.017 | .005 | .027 | .045 | 1 |  |  |
| **p*<.05; ***p*<.01; ****p*<.001; ^1^Gender (0=female, 1=male); ^2^Ethnicity (Arab=0, Jewish=1); ^3^Secular (1=secular; all else=0); ^4^Traditional (1=traditional; all else=0); ^5^Religious (1= religious; all else=0); ^6^Vaccination (0= haven’t been vaccinated, 1=vaccinated); ^7^Violator Gender (0=female, 1=male); ^8^Violator Ethnicity (Arab=0, Jewish=1); ^9^Secular Violator (1=secular; all else=0) ^10^Orthodox Violator (1=orthodox; all else=0); ^11^Funeral (participated in multi-participant funeral=1, all else=0); ^12^Wedding (participated in multi-participant wedding=1, all else=0); ^13^Protest (participated in multi-participant protest=1, all else=0); ^14^Confirmed COVID-19 (misbehavior involving a diagnosis of COVID-19 or contact with confirmed case of COVID-19=1, all else=0). | | | | | | | | | | | | | | | | | | |
